# Dogs learn to solve the support problem based on perceptual cues

**DOI:** 10.1007/s10071-014-0739-y

**Published:** 2014-03-04

**Authors:** Corsin A. Müller, Stefanie Riemer, Zsófia Virányi, Ludwig Huber, Friederike Range

**Affiliations:** 1 Messerli Research Institute, University of Veterinary Medicine Vienna, Medical University of Vienna, University of Vienna, Veterinärplatz 1, 1210 Vienna, Austria; 2 Department of Cognitive Biology, University of Vienna, Althanstrasse 14, 1090 Vienna, Austria

**Keywords:** Physical cognition, Means-end tasks, Problem solving, Domestic dogs, *Canis familiaris*

## Abstract

Numerous recent studies have investigated how animals solve means-end tasks and unraveled considerable variation in strategies used by different species. Domestic dogs (*Canis familiaris*) have typically performed comparably poorly in physical cognition tasks, but a recent study showed that they can solve the *on*–*off* condition of the support problem, where they are confronted with two boards, one with a reward placed on it and the other with a reward placed next to it. To explore which strategies dogs use to solve this task, we first tested 37 dogs with the *on*–*off* condition tested previously and then tested subjects that passed this condition with three transfer tasks. For the *contact* condition, the inaccessible reward was touching the second board. For the *perceptual containment* condition, the inaccessible reward was surrounded on three sides by the second board, but not supported by it, whereas for the *gap* condition, discontinuous boards were used. Unlike in the previous study, our subjects did not perform above chance level in the initial trials of the *on*–*off* condition, but 13 subjects learned to solve it. Their performance in the transfer tasks suggests that dogs can learn to solve the support problem based on perceptual cues, that they can quickly adopt new cues when old ones become unreliable, but also that some apparently inherent preferences are hard to overcome. Our study contributes to accumulating evidence demonstrating that animals typically rely on a variety of perceptual cues to solve physical cognition tasks, without developing an understanding of the underlying causal structure.

**Electronic supplementary material:**

The online version of this article (doi:10.1007/s10071-014-0739-y) contains supplementary material, which is available to authorized users.

## Introduction

Most humans can spontaneously solve means-end tasks by assessing the causal structure of the problem, without the need to resort to trial-and-error learning. This ability is based on “folk physics”, an understanding of the physical world that develops naturally in human infants and is built on observation of and experimentation with regularities in the physical world, which extend and/or confirm innate predispositions or core beliefs (Baillargeon 1994; Povinelli 2000; Spelke 2000; Baillargeon 2002; Carey 2009; Johnson 2010). In the last decades, considerable effort has been invested to investigate how animals solve such tasks. While claims of evidence for insightful problem solving in animals have typically not withstood scrutiny (c.f. Kacelnik 2009; Taylor and Gray 2009; Taylor et al. 2012), a large body of evidence has accumulated suggesting considerable variation between species in how they solve physical problems. A series of not mutually exclusive strategies have been suggested, which differ mainly in the extent to which they do (or do not) involve causal information in addition to reliance on learned perceptual cues. Among others, these include the development of an intuitive understanding through experience (Auersperg et al. 2009), a causality bias during associative learning (Hanus and Call 2011), reliance on perceptual correlates of the causal mechanism (Povinelli 2000) or heuristic strategies (Hunt et al. 2006), but also reliance on perceptual feedback (e.g., Taylor et al. 2012; Riemer et al. 2013) or simple trial-and-error learning with subsequent generalization (e.g., De Mendonça-Furtado and Ottoni 2008; Müller 2010).

Piaget’s support problem (Piaget 1952) has been used in a variety of species to test for their understanding of means-end connections (e.g., great apes: Povinelli et al. 2000; Herrmann et al. 2008; monkeys: Hauser et al. 1999; Yocom and Boysen 2010; Yamazaki et al. 2011; elephants: Irie-Sugimoto et al. 2008; and kea: Auersperg et al. 2009). It involves a target object (usually a piece of food) that is placed out of reach of the subject on a support (e.g., a piece of cloth or a wooden board) that is within the subject’s reach. In the classic setup, the subjects are presented with two choices: a support that carries a piece of reward, and a second support beside which another piece of reward has been placed. Subjects may solve this task spontaneously (e.g., Povinelli et al. 2000; Herrmann et al. 2008; Auersperg et al. 2009; Yamazaki et al. 2011) or learn to solve it after a number of sessions (e.g., Hauser et al. 1999; Irie-Sugimoto et al. 2008; Yocom and Boysen 2010). In either case, however, it remains unclear whether the subjects understood the causal structure of the task, or whether they solved the task based on perceptual cues such as contact between reward and support or perceptual containment of the reward within the support.

To determine whether subjects relied on perceptual cues to solve the classic support problem, and if so, which ones they used, it is necessary that successful subjects are subsequently presented with modified versions of the classic setup (transfer tasks) where causally relevant aspects of the setup have been changed. When employed previously, performance in such transfer tasks typically indicated that animals relied on a variety of perceptual cues to solve the classic support problem and consequently failed at least initially in one or several of the transfer tasks (Povinelli et al. 2000; Irie-Sugimoto et al. 2008; Auersperg et al. 2009; Yamazaki et al. 2011). For example, the chimpanzees tested by Povinelli et al. (2000, p. 268f) failed in transfer tasks where the reward was surrounded, but not supported by the cloth and thus may have relied on “current or imminent contact” for their choices, though this finding was not replicated in a later study with enculturated chimpanzees (Yocom and Boysen 2011). Also, the marmosets tested by Yamazaki et al. (2011) relied on several perceptual cues, including size of the reward, distance to the reward and distance between the support and the “off” reward. Similarly, the majority of the rooks that solved a different physical cognition task, the trap-tube task, did so by avoiding a perceptual cue, that is by pushing the reward away from the trap protruding from the tube (Tebbich et al. 2007) or from the black disk at the bottom of the functional trap (Seed et al. 2006).

Domestic dogs (*Canis familiaris*) have typically performed poorly in physical cognition tasks compared to other mammals (e.g., Collier-Baker et al. 2004; Osthaus et al. 2005; Bräuer et al. 2006; Fiset and Leblanc 2007). For example, in a two-choice task, dogs preferentially chose the container from which a noise had emanated both when the container was shaken and the piece of food in the container caused the noise, and when a cellular phone rang inside the container (a non-causal, arbitrary cue) (Bräuer et al. 2006). Also, dogs typically made their choice based on proximity rather than connectivity in string-pulling tasks (Osthaus et al. 2005; but see Riemer et al. 2013 for some exceptions). In contrast, a recent study (Range et al. 2011) suggested that dogs can perform well when presented with the support problem, where misleading proximity cues are less prominent than, for example, in the classic string-pulling tasks tested by Osthaus et al. (2005). In the study of Range et al. (2011), the dogs performed above chance level in four conditions where an out-of-reach food reward was placed on a board and a second reward was placed in different positions beside or behind a second board, so that the accessible reward was either closer, at equal distance or further away from the dog than the inaccessible reward.

The study of Range et al. (2011) suggests that dogs can solve the support problem, but it did not test which strategies they use to solve it. In particular, it remains unclear whether they solved the task based on perceptual cues, and if so, which cues they relied on. Here, we extend the study of Range et al. (2011) to determine which information dogs use to solve the support problem. After a replication of the classic *on*–*off* condition (condition 1 in Range et al. 2011; cf. Fig. 2), we tested successful dogs with three transfer tasks, the conditions *contact*, *perceptual containment* and *gap* (Fig. 2). With these, we tested whether the dogs that had solved the *on*–*off* condition had relied on particular visual cues when making their decision which option to choose to gain access to the out-of-reach reward.

Visual cues that could potentially be used when learning to solve the *on*–*off* condition include the color or brightness of the background on which the reward is resting, alignment of the reward and the board, contact between the reward and the board, perceptual containment of the reward within the board, continuity of the board and the reward, and the vertical level of the reward. Different predictions for the performance in the three transfer conditions, relative to the performance in the *on*–*off* condition, are made depending on which of these cues had been used to solve the *on*–*off* condition (summarized in Table 1). If the subjects learned to choose the side where the reward was resting on a bright yellow background (the color of the boards used in this study), rather than on a black background, we would expect their performance to remain on the same, high level in the *perceptual containment* condition, where this cue is still reliable. However, we would expect the performance to drop in the *contact* condition, where this cue is less obvious, and to drop to chance level in the *gap* condition, where the background cue is not informative. If the subjects learned to choose the reward that was aligned with the board, rather than the one that was misaligned, we would predict that performance drops to chance level in the *perceptual containment* condition and the *gap* condition, where alignment does not differ between the two options. Moreover, in this case performance should also be lower in the *contact* condition than in the *on*–*off* condition, since the alignment cue is less clear in the former than in the latter. If the subjects learned to choose the board that was in visual contact with the reward, we would predict that the performance drops to chance level in the *contact* condition and in the *gap* condition, where both rewards are in contact with one of the boards, but that it is not significantly reduced in the *perceptual containment* condition where, like in the *on*–*off* condition, the inaccessible reward does not touch the yellow board. If the subjects learned to choose the side of the reward that was visually surrounded by one of the boards (or perceptually contained within its optical field, cf. Povinelli et al. 2000; Auersperg et al. 2009), we would predict that performance would drop to chance level in the *gap* condition, where visual containment is equal for both options, and to drop also in the *contact* and *perceptual containment* conditions, since the difference in visual containment between the two options is reduced in both of them compared with the *on*–*off* condition. If the subjects learned to choose the board that provided an uninterrupted connection to one of the rewards, we would predict that the performance drops to chance level in the *contact* condition, where this is the case for both of the presented options. In contrast, we would predict that performance remains on a high level for the *gap* condition and that performance is reduced in the *perceptual containment* condition, where the gap between the board and the inaccessible reward is smaller than in the *on*–*off* condition. If the subjects learned to choose the side where the reward was presented on a higher level, we would predict that the performance remains equally high in the *contact* condition as in the *on*–*off* condition since in both of these, the accessible reward is presented on a higher level than the inaccessible reward. Performance in the *perceptual containment* and the *gap* conditions, however, is predicted to drop to chance level in that case, since in these conditions, both rewards are presented on the same vertical level. In contrast, if the subjects acquired an understanding of the underlying causal structure of the task, we would predict that performance does not drop significantly in any of the three transfer conditions when compared to the *on*–*off* condition.

**Table 1:** Perceptual cues that could be used to solve the *on*–*off* condition and corresponding predictions for performance in the three transfer conditions when compared to the performance in the *on*–*off* condition

Cue	Condition		
	Contact	Perceptual containment	Gap
Color or brightness of the reward’s background	Reduced	No change	Drop to chance level
Alignment of reward with board	Reduced	Drop to chance level	Drop to chance level
Contact between reward and board	Drop to chance level	No change	Drop to chance level
Perceptual containment of reward within board	Reduced	Reduced	Drop to chance level
Continuity of board and reward	Drop to chance level	Reduced or no change	No change
Vertical level of reward	No change	Drop to chance level	Drop to chance level

## Methods

### Subjects

We tested 37 Border Collies at the age of between 18 and 27 months (16 males, 21 females). Twenty-four of these subjects had been tested with a different physical cognition task, the string-pulling problem, before (Riemer et al. 2013). All subjects lived as pet dogs with their owners, who volunteered to bring their dogs to the Clever Dog Lab for this study. We tested dogs of a single breed with the aim of reducing variability induced by breed differences and chose Border Collies due to their high availability and motivation to work with humans. Also, this breed is neither highly brachycephalic nor highly dolichocephalic (characteristics that may provide advantages or disadvantages in visual tasks; McGreevy et al. 2004; Gácsi et al. 2009), and we have no reason to assume that Border Collies were selected for performance in means-end tasks.

### Apparatus and conditions

**Fig. 1:**
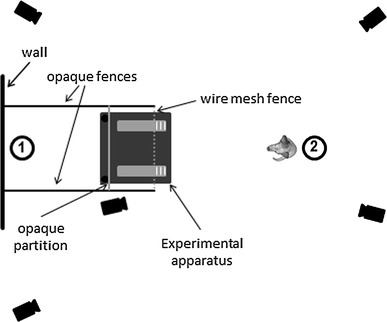
Layout of the experimental setup. *Circles* indicate positions of E1 and E2, respectively

Testing took place in a 5 by 6 m room at the Clever Dog Lab in Vienna, Austria. The test apparatus consisted of two yellow wooden boards (11 by 60 by 2 cm) mounted on a 90 by 90 cm black platform (distance between boards: 40 cm). They could be moved backward and forward on rails embedded in the platform. Three wooden strips were fixed to the proximal end of both boards to ensure that the dog could find purchase when trying to pull them out. The apparatus was placed inside a 1 by 2 m fenced area with opaque sides and a wire mesh front, from where it was operated by experimenter 1 (E1). E1 could push out the apparatus through a 5 cm gap at the bottom of the front fence at the beginning of each trial, and pull it back in at the end of the trial (Fig. 1, see also supplementary videos). An opaque partition mounted 50 cm behind the front fence prevented visual contact between the dog and E1 and prevented the dog from observing the baiting process. This modification compared with the setup of Range et al. (2011) was introduced to address the possibility that the dogs may have preferentially chosen the positive option, the board with the obtainable reward, because it presented the same layout as the one seen (and rewarded) during pre-trials in the 2011 study, and to exclude any possible unconscious cueing by E1. A camera was set up next to the fenced area so that E1 could see, on the camera screen, when the dog was ready and a trial could start (Fig. 1).

**Fig. 2:**
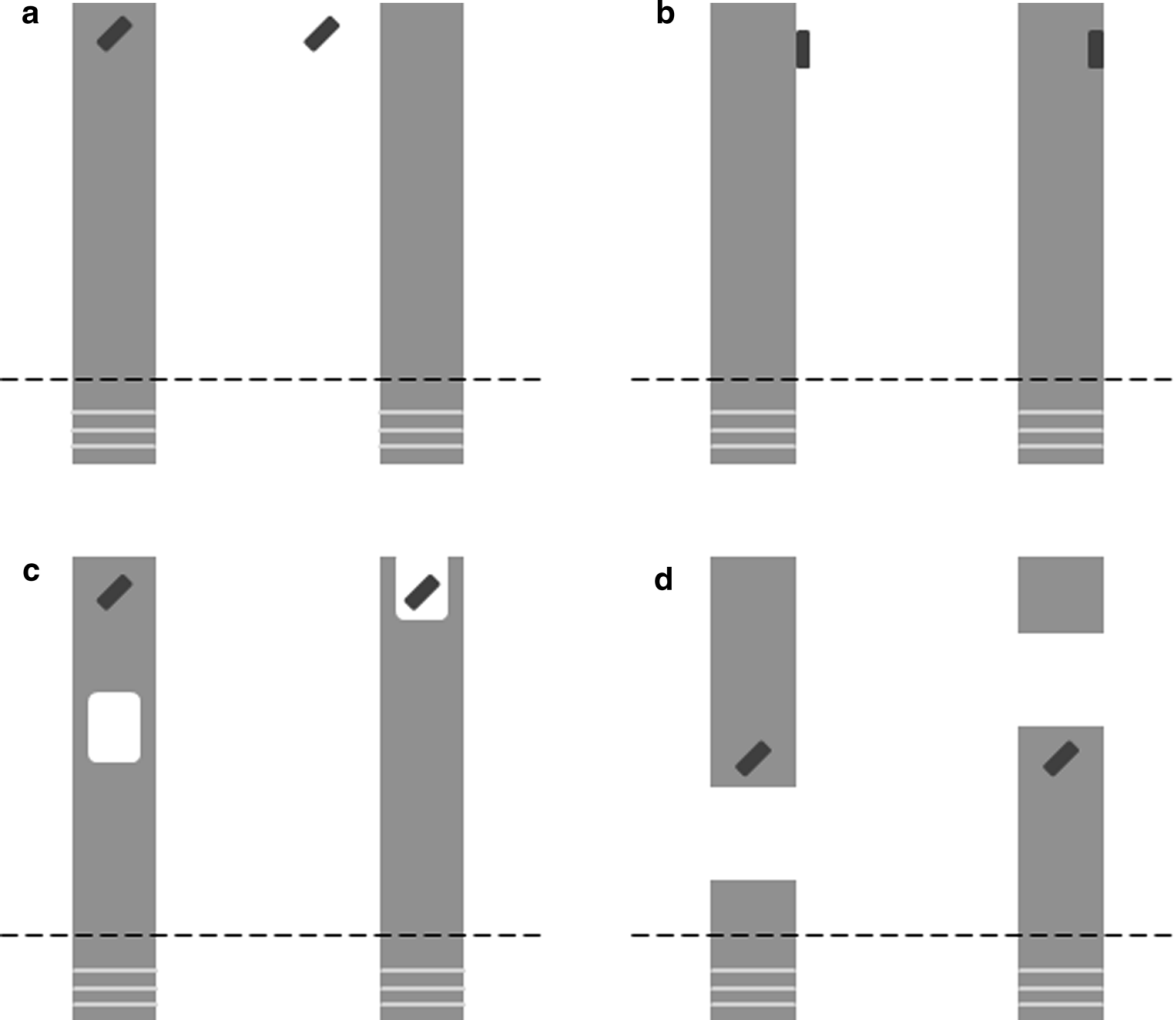
Illustration of the experimental apparatus in the four conditions used in the experiments when seen from above: *on*–*off* (**a**), *contact* (**b**), *perceptual containment* (**c**) and *gap* (**d**). The *dashed line* indicates the location of the wire mesh fence separating the dog’s area (*bottom*) from the compartment of E1. Note that for the *perceptual containment* condition and the *gap* condition, the inaccessible reward was presented on the same level above ground as the accessible reward, whereas the same was not the case for the *on*–*off* and the *contact* condition

Four different conditions were presented to the dogs, with 4 cm strips of sausage used as rewards (Fig. 2). In all conditions, the two rewards were placed at equal distance from the subject. For the *on*–*off* condition, a reward was placed on one of the boards, and another reward was placed 5 cm beside the other board (randomly on the left or on the right side of it). This condition replicated condition 1 of the Range et al. (2011) study. For the *contact* condition, a reward was placed next to and touching one of the boards, whereas the second reward was placed on the top edge of the other board (Fig. 2). Again, the two rewards were placed randomly, either both on the right or both on the left side of the corresponding board. For the *perceptual containment* condition, we used two boards with 7 cm wide and 10 cm long cut out areas in different positions (Fig. 2). We introduced a cut out in both boards to ensure that both options looked different from the layout of the rewarded option in the *on*–*off* condition. Likewise, for the *gap* condition, a 12 cm gap in the board was introduced either in front of or behind the position of the reward (Fig. 2).

### Procedure

Before testing started, the dogs were trained to pull out the boards using a shaping procedure. For this purpose, the front fence was removed, so that the dogs could walk up to the opaque partition behind it. A single baited board was pushed out (pseudorandomly on the left or on the right side) until the front part of the board (ca 12 cm) became accessible to the dog (cf. supplementary video). A reward was initially placed on the board just behind the partition, so that the dog could smell, but not see it. When the dog successfully obtained the reward by pulling out the board with its paws, the distance between the reward and the partition was increased stepwise in subsequent shaping trials. Once a dog retrieved the reward placed at the furthest distance four times in a row (twice on each side), it proceeded to testing. Note that due to the opaque partition, the dogs never saw the layout that would be positive during subsequent testing in this phase, unlike in the training phase of the 2011 study (Range et al. 2011).

For each test trial, experimenter 2 (E2) brought the dog to a position 1.5 m in front of the wire mesh fence (cf. Fig. 1) and put on a blindfold. E1 then started the trial by pushing out the test apparatus so that its front became accessible to the dog. When E1 saw on the screen of the camera that the dog had looked at the apparatus for 5 s, E1 knocked on the ground between the two boards, signaling to E2 to release the dog. E2 released the dog upon the signal, additionally giving a verbal “go” command for dogs that did not leave of their own accord. Once the dog had left the start position, E2 removed the blindfold and retrieved the dog when it had obtained the reward after pulling out the correct board or when it had pulled out the incorrect board at least half way (see also supplementary videos). The owner remained outside the testing room throughout the test trials.

The dogs received 2–4 (median 3) test sessions of 10 trials per day, with a break of at least 5 min between sessions, during which dog and experimenters left the testing room. Test days were separated by a median of 7 days (range 1–37, depending on availability of the dogs). The correct side was varied pseudorandomly so that the same side was correct never more than twice in a row. All dogs were first tested with the *on*–*off* condition for a maximum of 6 sessions (the acquisition phase). Dogs that passed the learning criterion of at least 16 correct choices in two consecutive sessions (20 trials) or at least 22 correct choices in three consecutive sessions (30 trials; binomial probability <0.02) were subsequently tested with 48 intermixed trials of the four conditions (12 trials per condition in randomized order).

### Analyses

For each trial, two variables were coded: (1) which board the dog touched first and (2) which board the dog pulled out first by at least 20 cm (half way to obtain the reward). A correct choice was coded only if the dog touched the correct board first *and* pulled it out first. Note that, for the first touch, it was possible but not required that the board moves by a few centimeters. Coding was done from video recordings, with the exception of 23 trials (of the total 2,464) where due to equipment failure we used data from notes taken by E1 during the experiment. Concordance between notes and video coding was high [99.5 % for first touch, 100 % for first pull based on 20 randomly chosen sessions (200 trials)]. Reliability coding from video recordings was done by a coder who was unfamiliar with the goals of the study for 20 randomly chosen sessions and reliability was excellent (99.5 % for first touch, 100 % for first pull).

All analyses were performed in R 2.15.1 (R Core Team 2012). The choice data were analyzed with binomial generalized linear models (GLMs) with logit link, with the number of correct choices in the numerator and the number of trials in the denominator of the response variable. GLMs were run with correction for over-dispersion if applicable. We used generalized linear mixed models (GLMMs) with dog identity included as a random effect to test for differences between conditions or sessions and for learning across trials (using R package lme4, Bates et al. 2012). To determine whether the correct choice probability differed from chance level for particular sessions or conditions, we tested whether the intercept differed from 0 (=log(1), corresponding to the chance level of 50 %) in binomial GLMs with the intercept as the only predictor. Subject sex was initially included in all models as a predictor, but dropped in all cases as non-significant. The 24 subjects with string-pulling experience did not perform better in the initial session of the *on*–*off* condition (binomial glm: *z* = 0.07, *p* = 0.94) and were not more likely to pass the *on*–*off* condition (Fisher’s exact test: *p* = 1) than subjects without string-pulling experience. Also, the performance in the intermixed trials was not affected by string-pulling experience (GLMM, *z* = −0.27, *p* = 0.78). The data of the two groups were therefore pooled for all analyses.

### Ethical note

The experiments and procedures presented in this manuscript adhered to the “guidelines for the treatment of animals in behavioral research and teaching” as published by the ASAB (2006) and are in accordance with the Austrian Federal Act on the Protection of Animals (Animal Protection Act—TSchG, BGBl. I Nr. 118/2004). Furthermore, as the present study was strictly non-invasive, no special permission was required in accordance with the Austrian Animal Experiments Act (§ 2, Federal Law Gazette No. 501/1989).

## Results

### Acquisition phase: on–off condition

**Fig. 3:**
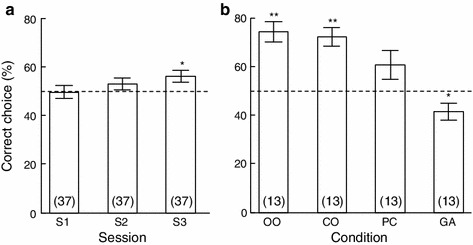
Percent correct choices for the first three sessions of the *on*–*off* condition (**a**) and for the four conditions during intermixed trials (**b**). *OO* *on*–*off* condition, *CO* *contact* condition, *PC* *perceptual containment* condition, *GA* *gap* condition. *Numbers* in *parentheses* give sample sizes. The *dashed lines* indicate chance level. Data are displayed as mean and standard error. *Stars* indicate significant deviation from chance level: *p < 0.05; **p < 0.001

Group performance in the initial 10 trials did not differ from chance (Table 2; GLM: *z* = −0.01, *p* = 0.96), but performance improved significantly across sessions (GLMM: *z* = 4.78, *p* < 0.001) and was significantly above chance level in the 10 trials of the third session (GLM: *z* = 2.39, *p* = 0.017; Table 2; Fig. 3a). Improvement between adjacent sessions that took place on different test days was not affected by the interval between the two test days (session-by-interval interaction, GLMM: *z* = 0.14, *p* = 0.89). Also, whether the *off* reward was placed on the inside of the second board (i.e., between the two boards as shown in Fig. 2) or on the outside did not affect the probability of a correct choice (GLMM: *z* = 1.03, *p* = 0.30). No dog reached the individual-learning criterion at the earliest opportunity (after 2 sessions), but 13 of the 37 subjects reached the individual-learning criterion after 3–6 sessions (median: 5 sessions).

**Table 2:** Proportion of correct trials across sessions of the acquisition phase

Session	Mean	Standard error	N^a^
1	0.498	0.026	37
2	0.531	0.025	37
3	0.562	0.025	37
4	0.603	0.028	36
5	0.633	0.029	33
6^b^	0.767	0.033	3

^a^The sample size decreases across sessions as subjects that reached the learning criterion moved on to the transfer tests (intermixed trials)

^b^Only dogs that still had a chance of reaching the learning criterion were tested in the last session

Switching to the other board after one board had been touched (without pulling it out at least half way) occurred in 4.5 % of all trials. Switching was significantly more likely to happen after a first touch to the incorrect board than after a first touch to the correct board (7.9 and 2.0 % of all trials, respectively, GLMM: *z* = −5.38, *p* < 0.001), indicating that at least some dogs occasionally used visual feedback for their decision about which board to pull out. Sixteen of the 37 dogs switched between boards in more than one trial. Overall, these “switchers” made fewer correct choices than the other individuals (GLMM: *z* = −2.40, *p* = 0.017), which might appear surprising given that they showed more flexibility than the other dogs. However, this result is probably merely a side effect of our definition of a correct choice, which required that the subject does not switch boards within a trial for a correct choice to be coded. Correspondingly, when trials in which switching occurred were excluded from this analysis, the effect disappeared (GLMM: *z* = −0.85, *p* = 0.39).

### Four conditions intermixed

For the 13 subjects tested with the four conditions intermixed, performance differed significantly between conditions (GLMM with likelihood ratio test, *χ*_(3)_^2^ = 45.7, *p* < 0.001; Fig. 3b). In *on*–*off* trials, group performance was significantly above chance (74.5 % correct, GLM: *t*_(12)_ = 4.88, *p* < 0.001) and not significantly lower than the performance of the same thirteen subjects in their last session of the acquisition phase (80.8 % correct, GLMM: *z* = 1.26, *p* = 0.21). Six subjects performed significantly above chance at the individual level (≥10 of 12 trials correct, binomial test: *p* < 0.04). That only half of the subjects reached the individual-level criterion again is probably explained by two factors: First, the criterion was more difficult to reach since fewer trials were performed and second, the presentation of different conditions in intermixed order likely led to increased error rates as subjects were trying to determine alternative cues that help them to solve the new conditions.

For *contact* trials, group performance was also above chance (72.3 % correct, GLM: *t*_(12)_ = 4.87, *p* < 0.001), and not significantly different from the performance in *on*–*off* trials (GLMM: *z* = −0.45, *p* = 0.65). However, only three subjects performed significantly above chance at the individual level; one of them was among the six subjects that performed significantly above chance in the *on*–*off* trials. In *perceptual containment* trials, group performance was not significantly above chance level (60.9 % correct, GLM: *t*_(12)_ = 1.78, *p* = 0.10) and significantly lower than for *contact* trials (GLMM: *z* = −2.15, *p* = 0.03). Three subjects performed significantly above chance at the individual level in containment trials; these were not the same subjects that reached the criterion for the *contact* condition. In *gap* trials, group performance was significantly below chance level (41.7 % correct, GLM: *t*_(12)_ = −2.32, *p* = 0.04); no subject performed significantly different from chance at the individual level in this condition.

There was some evidence for learning across trials, reflected in a significant condition-by-trial interaction (GLMM with likelihood ratio test: *χ*_(3)_^2^ = 11.2, *p* = 0.01). For the *on*–*off* condition, performance decreased across trials in the intermixed phase (*z* = −2.14, *p* = 0.03). In contrast, performance improved significantly across trials for the *perceptual containment* condition (GLMM: *z* = 2.69, *p* = 0.007) but not for the *contact* condition (*z* = 1.08, *p* = 0.28) or for the *gap* condition (*z* = 0.84, *p* = 0.40). The improvement across the twelve trials of the *perceptual containment* condition was steeper than the improvement the same thirteen individuals had shown across their first twelve trials of the *on*–*off* condition in the acquisition phase, though this effect did not reach significance (condition-by-trial interaction: *z* = 1.74, *p* = 0.08).

## Discussion

A third of the tested dogs learned to solve the *on*–*off* condition of the support problem within a maximum of 60 trials. These dogs subsequently also solved the transfer condition where the off reward was also touching the support (*contact* condition) and showed evidence for quick learning within 12 trials of the *perceptual containment* condition. In contrast, the dogs consistently failed in the *gap* condition, where rewards were presented on discontinuous supports.

Performance in the first session of the *on*–*off* condition was not different from chance level, unlike in the earlier study of Range et al. (2011) where the dogs performed above chance level in the same condition with only 12 trials per subject. This discrepancy cannot be explained by the use of different subjects, since performance in the Range et al. (2011) study was still significantly above chance when their sample was restricted to Border Collies (*N* = 10) or to dogs between 1 and 2 years of age (*N* = 8). The discrepancy also cannot be explained by a different criterion for correct choices, since the dogs in the present study also did not perform above chance level in their first session when the criterion of the Range et al. (2011) study was used (first touch to the correct board without need to pull it out on their own). Finally, one might suspect that the dogs in the Range et al. (2011) study, which were tested with a total of 48 trials of four variations of the *on*–*off* condition in intermixed order, may have reached criterion because they showed learning across conditions and thus performed well particularly in the second half of the trials. However, this suggestion is not supported since the dogs in the Range et al. (2011) study performed significantly above chance level already in the first six trials and showed no evidence of learning across trials of the condition corresponding to the *on*–*off* condition in the present study (Range et al. 2011).

We suggest that the methodological change introduced in this study, the opaque partition between dog and experimenter, is responsible for the diverging results of the present and the previous study (Range et al. 2011). In particular, the dogs in the 2011 study may have chosen the correct board in the *on*–*off* condition because it presented the same layout they had already seen (and which was already rewarded) during their training phase, during which only one (the positive, rewarded) option was presented and the subjects learned to perform the action necessary to pull out the board (in a mean of 22 training trials; Range et al. 2011). In contrast, in the present study, visual access to the layout during the training phase was precluded by an opaque partition. That is, the dogs in the 2011 study may have already started to associate the positive layout with being rewarded in the training session, whereas the dogs in the present study could start learning to recognize the positive layout only once the test trials of the *on*–*off* condition were presented. This line of argument is similar to the one made by Povinelli et al. (2000), who found some indication that the performance of chimpanzees in support tasks was improved if one of the options presented matched the layout known from earlier conditions.

The learning performance of dogs in the *on*–*off* condition lies within the range of performances found for other species. While the two Asian elephants tested by Irie-Sugimoto et al. (2008) needed between 120 and 240 trials to reach a criterion comparable to the one used by us, in chimpanzees (Povinelli et al. 2000) and kea (Auersperg et al. 2009), the majority of subjects solved the *on*–*off* condition much more quickly (within 8–10 trials). Note though that the training procedure used by Auersperg et al. (2009), similarly to Range et al. (2011), presented the later positive “on” layout already during the training trials and, as suggested by our results, this may have contributed to the excellent performance of the kea in the first trials of the *on*–*off* condition.

Like chimpanzees (Povinelli et al. 2000) and marmosets (Yamazaki et al. 2011), the dogs in our study showed little difficulty with the *contact* condition. Indeed, the dogs performed at a similar level of accuracy in the *contact* condition as in the *on*–*off* condition, which stands in contrast to the primate data. The chimpanzees tested by Povinelli et al. (2000) performed worse in the *contact* condition, though the effect for the eight subjects did not reach significance (see also Yocom and Boysen 2011). The four marmosets tested by Yamazaki et al. (2011) performed significantly worse in the *contact* condition (the “standard condition” in their terminology) than in the *on*–*off* condition (the “longer distance condition” in their terminology) as revealed by our re-analysis of the data presented in their Fig. 2 (pooling the six variations of each condition; binomial GLMM with subject identity as a random factor and condition as a predictor: *z* = 4.11, *p* < 0.001).

In the *perceptual containment* condition, the performance of the dogs dropped to chance level at first, but quickly and significantly improved thereafter. The initial problems of the dogs with transferring from the *on*–*off* to the *perceptual containment* condition match the findings of Povinelli et al. (2000) for chimpanzees, which performed poorly in the *perceptual containment* conditions, a finding that led them to suggest that the apes may rely on current or imminent contact between reward and support when attempting to solve the support problem (but see Yocom and Boysen 2011). In contrast, the keas tested by Auersperg et al. (2009) performed above chance level in the *perceptual containment* conditions from their first session on, which, as they suggested, may be explained by the superior visual acuity of birds compared with mammals.

The *gap* condition has consistently proven to be among the most challenging of the support problem conditions presented to animals so far. In addition to the dogs in our study, pigeons (Schmidt and Cook 2006), tamarins (Hauser et al. 1999), marmosets (Yamazaki et al. 2011) and an Asian elephant (Irie-Sugimoto et al. 2008) have either failed in this task or required a considerable number of trials to learn to solve it, while kea appeared to learn more quickly and one individual reached criterion within ten trials (Auersperg et al. 2009). Only for great apes, there is some evidence that they can perform above chance level spontaneously in this condition, though at a low level of accuracy of less than 60 % correct choices (Herrmann et al. 2008). Unlike other species tested so far, however, the dogs in our study performed significantly below chance level in the *gap* condition, thus showing a preference for the shorter, non-rewarded board. This finding matches the one of a recent string-pulling study, in which dogs also developed a preference for the shorter, non-rewarded string over a longer rewarded string (Range et al. 2012). While these unexpected results remain unexplained, the replication within species and the contrast to other species suggest that they may reflect a species-specific predisposition, for example a preference for a smaller over a larger non-food object that needs to be handled or moved.

The variable performance of the dogs across conditions suggests that they used a perceptual cue (or a combination of cues) in their attempts to solve the support problem. The combination of an excellent performance in the *contact* trials, which was statistically not different from the performance in the on–off trials, and the poor performance in the *perceptual containment* and *gap* trials is most consistent with the suggestion that many of the dogs had learned to choose the reward that was on the board rather than the one that was off the other board and thus on a lower level. (cf. Table 1). The dogs did not appear to use the color or brightness of the background on which the reward was resting (bright yellow for the reward on, black for the reward off the support) to guide their choices. In that case, transfer to the *perceptual containment* condition should have been instantaneous. The dogs’ good performance in the *contact* condition also indicates that they had not learned to base their choices on the visual contact between reward and board or based on the alignment of board and reward. Finally, also a reliance on perceptual containment does not appear to be consistent with our results as, in this case, we would have predicted a reduced performance in the *contact* condition (where containment was similar between the two options) compared with the *on*–*off* condition (where the accessible reward was surrounded on all four sides by the support, and the inaccessible reward was not contained at all).

Our finding that the dogs showed quick learning in the *perceptual containment* condition, with a parallel decrease in performance in the *on*–*off condition*, also indicates that perceptual cues may be quickly abandoned once they became unreliable. In contrast the poor, below chance performance in the *gap* condition indicates that some inherent biases are hard to overcome in such perceptual learning tasks (see also Head et al. 1998; Kelber 2002; Miller and Pawlik 2013). Finally, the exceptional performance of three subjects in the *perceptual containment* condition, in contrast to the group-level performance for this condition which was initially at chance level, suggests that these individuals may have relied on a different perceptual cue, such as the yellow versus black background in which the rewards were presented, to solve the support problem.

Rather than making their choice based on a perceptual cue of the presented layout, the subjects could have made their decision which board to pull out based on visual feedback, as previously found in string-pulling tasks for corvids (Taylor et al. 2012) as well as for some dogs (Riemer et al. 2013). That is, the subjects could have used the strategy to pull out one support a bit and then decide whether to continue or switch to the other option based on whether the reward moved or not. However, this strategy was used only rarely by the dogs in the present study (note that, following our criterion, these trials were coded as incorrect choices, even though the dogs obtained the reward).

To conclude, our study shows that at least some dogs can learn to solve the classic Piagetian support problem, but appear to do so by associating perceptual cues that are not causally related to the physical underpinnings of the task with the obtaining of a reward. The subjects that had solved this problem subsequently showed significant transfer to the condition where the perceptual cue was still reliable and some evidence of quick adoption of new cues when the originally learned cue was no longer reliable. Our results thus add to a growing body of evidence that animals typically rely on, or learn to attend to, perceptual cues that may be correlated with the causally relevant information, but are not representing the underlying causal structure, when facing a problem-solving task (cf. Povinelli 2000; Penn and Povinelli 2007; Yamazaki et al. 2011; Albiach-Serrano et al. 2012; Gajdon et al. 2013). In addition, our data suggest that associative learning of perceptual cues may start already during training or habituation phases and that initial performance in previous studies may thus have been overestimated. Based on this finding, it is recommended that the positive (or negative) layout used during testing should not be presented in pre-trials of physical cognition tasks, during which subjects commonly get habituated to the apparatus and learn to perform the necessary actions. The considerable variation in performance we found between individuals furthermore calls for an in-depth analysis of the factors affecting individual performance in cognitive tasks (see also, e.g., Herrmann and Call 2012).

## Electronic supplementary material

Below is the link to the electronic supplementary material.

Supplementary material 1 (PDF 44 kb)

Supplementary material 2 (MPG 1366 kb)

Supplementary material 3 (MPG 2332 kb)

Supplementary material 4 (MPG 2262 kb)

Supplementary material 5 (MPG 3164 kb)

Supplementary material 6 (XLSX 21 kb)
