# Supplementary material for: Dogs learn to solve the support problem based on perceptual cues
Source: Anim Cogn. 2014 Mar 4;17(5):1071–80. doi: 10.1007/s10071-014-0739-y (PMC4138433; doi:10.1007/s10071-014-0739-y)
Supplement: Supplementary file 1 — Supplementary material 1 (PDF 44 kb) [file 10071_2014_739_MOESM1_ESM.pdf]

Supplementary Material

Dogs learn to solve the support problem based on perceptual cues

Animal Cognition

Corsin A. Müller*, Stefanie Riemer, Zsófia Virányi, Ludwig Huber, Friederike Range

*corsin.mueller@vetmeduni.ac.at

1) Example video clips†

Supplementary Video 1: Shaping trial towards end of the shaping phase

Supplementary Video 2: Test trial, on-off condition, correct choice

Supplementary Video 3: Test trial, contact condition, incorrect choice

Supplementary Video 4: Test trial, perceptual containment condition - switch after incorrect first touch (considered as incorrect choice)

† may not play in Windows Media Player, use VLC player instead.

2) Dataset of proportion of correct choices, side bias and occurrence of switching for each session of the acquisition phase (on-off condition) and for the four conditions presented during intermixed trials.
